# Relationship Between Lifestyle Habits and Health-Related Quality of Life of Recently Diagnosed Breast Cancer Patients: A Comparison Between Younger and Older Women in China

**DOI:** 10.3389/fpubh.2021.767151

**Published:** 2021-12-14

**Authors:** Chao Zheng, Li-Xiang Yu, Hong-Ying Jia, Shu-De Cui, Fu-Guo Tian, Zhi-Min Fan, Cui-Zhi Geng, Xu-Chen Cao, Zhen-Lin Yang, Xiang Wang, Hong Liang, Shu Wang, Hong-Chuan Jiang, Xue-Ning Duan, Hai-Bo Wang, Guo-Lou Li, Qi-Tang Wang, Jian-Guo Zhang, Feng Jin, Jin-Hai Tang, Liang Li, Shi-Guang Zhu, Wen-Shu Zuo, Fei Wang, Fei Zhou, Yu-Juan Xiang, Ming-Ming Guo, Yong-Jiu Wang, Shu-Ya Huang, Li-Yuan Liu, Zhi-Gang Yu

**Affiliations:** ^1^Department of Breast Surgery, The Second Hospital, Cheeloo College of Medicine, Shandong University, Jinan, China; ^2^Institute of Translational Medicine of Breast Disease Prevention and Treatment, Shandong University, Jinan, China; ^3^Center of Evidence-Based Medicine, The Second Hospital, Cheeloo College of Medicine, Shandong University, Jinan, China; ^4^Department of Breast Surgery, Affiliated Tumor Hospital of Zhengzhou University, Zhengzhou, China; ^5^Department of Breast Surgery, Shanxi Cancer Hospital, Taiyuan, China; ^6^Department of Breast Surgery, The First Hospital of Jilin University, Changchun, Jilin, China; ^7^Breast Center, The Fourth Hospital of Hebei Medical University, Shijiazhuang, China; ^8^Department of Breast Surgery, Tianjin Medical University Cancer Institute and Hospital, Tianjin, China; ^9^Department of Thyroid and Breast Surgery, The First Affiliated Hospital of Binzhou Medical University, Binzhou, China; ^10^Department of Breast Surgery, Cancer Hospital, Chinese Academy of Medical Sciences, Beijing, China; ^11^Department of General Surgery, Linyi People's Hospital, Linyi, China; ^12^Breast Disease Center, Peking University People's Hospital, Beijing, China; ^13^Department of General Surgery, Beijing Chaoyang Hospital, Beijing, China; ^14^Breast Disease Center, Peking University First Hospital, Beijing, China; ^15^Breast Center, Qingdao University Affiliated Hospital, Qingdao, China; ^16^Department of Breast and Thyroid Surgery, Weifang Traditional Chinese Hospital, Weifang, China; ^17^Department of Breast Surgery, The Second Affiliated Hospital of Qingdao Medical College, Qingdao Central Hospital, Qingdao, China; ^18^Department of General Surgery, The Second Affiliated Hospital of Harbin Medical University, Harbin, China; ^19^Department of Breast Surgery, The First Affiliated Hospital of China Medical University, Shenyang, China; ^20^Department of General Surgery, Nanjing Medical University Affiliated Cancer Hospital, Cancer Institute of Jiangsu Province, Nanjing, China; ^21^Department of Breast and Thyroid Surgery, Zibo Central Hospital, Zibo, China; ^22^Department of Breast Surgery, Yantai Yuhuangding Hospital, Yantai, China; ^23^Breast Cancer Center, Shandong Cancer Hospital, Jinan, China

**Keywords:** quality of life, breast cancer, lifestyle habits, age-related differences, patient satisfaction, prognosis

## Abstract

**Objective:** The aim of this study was to evaluate the relationship between lifestyle habits and health-related quality of life (HRQoL) among different ages who were initially diagnosed with breast cancer (within the first 2 weeks) and to determine the contribution of lifestyle habits factors on HRQoL.

**Methods:** Patients with breast cancer were recruited from 22 hospitals in 11 provinces or municipalities in northern and eastern China. The Functional Assessment of Cancer Therapy-Breast Cancer (FACT-B) was used to measure HRQoL. Chi-square test, ANOVA, and multivariable generalized linear models were conducted to identify the differences in HRQoL between two age groups (age <50 years and ≥50 years) and to evaluate the contribution of lifestyle habits factors on HRQoL of patients with breast cancer.

**Results:** About 1,199 eligible patients with breast cancer were used for analysis. Younger women (aged <50 years) appeared to show lower scores than older women (aged ≥50 years) in HRQoL subscales, including emotional well-being (*p* = 0.003), functional well-being (*p* = 0.006), breast cancer subscale (*p* = 0.038), and FACT-B Total scores (*p* = 0.028). Tea and alcohol consumption and being very satisfied with sleep and current life were the strongest predictors of higher HRQoL in younger group. Meanwhile, no coffee consumption, frequent participation in physical activities, high sleep satisfaction, and current life satisfaction were the key predictors of higher HRQoL in older women with breast cancer.

**Conclusion:** The relationship of the nine lifestyle habit items with HRQoL differed among younger and older women. The associated variable of low HRQoL can help clinicians take intervention early in order to improve the prognosis of patients with breast cancer.

## Introduction

Breast cancer is the most common cancer among women in China. According to the data released by the National Cancer Center of China, in 2020, there were 4,16,371 new cases of breast cancer among women in China (1). As evidenced by data on breast cancer with high incidence rates and relatively low mortality rates, it is the most prevalent cancer in China (2). It has been predicted that by 2021, there will be 2.2 million cases of breast cancer in China among women aged 35–49 years in 2001, which is equivalent to more than 100 new cases per 1,00,000 women (3). Therefore, efforts to improve the health-related quality of life (HRQoL) of this growing population of women have thus become an issue of great public health importance (4–6).

In 1993, the World Health Organization (WHO) defined quality of life (QoL) as “the comprehensive satisfaction of individuals in different cultures and value systems with their goals, expectations, standards, and life conditions related to their concerns, as well as their general sense of personal health” (7). The QoL reflects the overall physical and mental response and the sense of real self-worth from the heart when the individual suffers from the pain (8). This functional scale for a certain cancer can accurately evaluate the patient's condition and play an irreplaceable role (9, 10), and is now considered an important endpoint in cancer clinical trials (5).

As well-documented in previous studies, evaluating the HRQoL of patients with cancer could contribute to improved treatment and could even predict prognosis as medical factors can predict prognosis (11–14). From a clinical point of view, patients with breast cancer perform poorly in terms of psychology, physiology, and sociology. Therefore, patients with breast cancer need more support and help in these aspects. It is very important to evaluate and study the HRQoL of patients with breast cancer, pay attention to their physical and mental condition, and take active measures and intervention methods to improve their HRQoL (6).

At present, many researchers are committed to exploring the influencing factors affecting the HRQoL of patients with breast cancer and hope to improve the overall health of patients with breast cancer by changing these factors. Psychosocial factors, sociodemographic variables, and medical variables have been identified as predictors of HRQoL in patients with cancer (10, 12), but current research on the impact of various lifestyle habits factors on the HRQoL of patients with breast cancer is still controversial (15, 16). Mosher and Danoff-Burg reviewed and analyzed the studies of age differences in breast cancer psychological adaptation and believed that age may be a risk factor for distress with other variables contributing to this demographic difference (17). Furthermore, previous studies have shown that younger women have a higher psychological incidence rate and poorer HRQoL after a breast cancer diagnosis than older women (4, 13, 18). However, the differences in the relationship between lifestyle habits and HRQoL in younger and older women in China have not been studied. More importantly, most studies on QoL after breast cancer are conducted at least 4 months to more than 5–10 years after women are diagnosed with cancer and sometimes after the completion of treatment (12, 13, 15). Few studies have evaluated the HRQoL of women initially diagnosed with breast cancer in the first few weeks (19).

The aim of this study was to evaluate the relationship of lifestyle habits with HRQoL among younger and older women who were initially diagnosed with breast cancer within the first 2 weeks and to determine the contribution of lifestyle habits factors on the HRQoL. The findings of this study can potentially help guide the training/initiatives that are organized for shaping lifestyle habits of patients with breast cancer after cancer diagnosis and may influence the future course and prognosis of breast cancer in women.

## Materials and Methods

### Recruitment

A multicenter, hospital-based case–control study was conducted among breast cancer women from 22 hospitals in 11 provinces or municipalities in northern and eastern China from April 2012 to April 2013, as described previously (20, 21). Of these, Han Chinese women newly diagnosed with primary breast cancer confirmed by histology and aged from 25 to 70 years were included in this subgroup study. Exclusion criteria were as follows: (1) being <25 or >70 years of age; (2) being diagnosed with recurrent breast cancer; (3) being diagnosed with metastatic breast cancer; (4) patients with other malignant tumors; and (5) patients who refused to enroll. A self-designed structured questionnaire was used on the interview, as described previously (20, 21). The questionnaire mainly includes the following contents: demographic characteristics, female physiological and reproductive factors, medical and family history, lifestyle habits, and breast cancer-related knowledge. In this study, we only analyzed the lifestyle habits, including smoking (including passive smoking), alcohol consumption, dietary habits (tea and coffee consumption), sleep satisfaction, current life satisfaction, physical activity, and body mass index (BMI, kg/m^2^).

### Assessment of HRQoL

The Functional Assessment of Cancer Therapy-Breast Cancer (FACT-B)-simplified Chinese version 4 instrument is administered during the baseline interview to assess HRQoL. The FACT-B is a 36-item questionnaire that includes both 27 items of general HRQoL (FACT-G) associated with cancer and another nine items of HRQoL related to breast cancer, the breast cancer subscale (BCS). FACT-B consists of the following subscales: physical well-being (PWB) (seven items), social/family well-being (SWB) (seven items), emotional well-being (EWB) (six items), functional well-being (FWB) (seven items), and BCS. The simplified Chinese translation was performed using a standardized methodology by a series of forward and backward translations as well as review and field testing. The FACT-B uses a 5-point scale (0 = not at all; 1 = a little bit; 2 = somewhat; 3 = quite a bit; and 4 = very much) to indicate how true the statements were to the subjects over the previous 7 days. If more than half of the items that make up the subscale were answered, the missing values were calculated as an average of the observed items. Depending on the scale, higher scores may represent either a higher level of well-being or a lower level of well-being. The items that are expressed in the opposite direction were transformed before being summed up to calculate each subscale's score. Higher scores represent higher levels of well-being. The Cronbach α in this study was 0.881 for the FACT-B Total, 0.821 for PWB, 0.800 for SWB, 0.757 for EWB, 0.876 for FWB, and 0.653 for BCS.

Before the start of this study, the investigators were all trained and assessed. The baseline and FACT-B questionnaires were collected in a unified standard and unified manner to reduce information bias. Face-to-face interviews were conducted to collect the basic information and HRQoL information from the patients. And the baseline and FACT-B questionnaires were completed within 2 weeks after the diagnosis of breast cancer; none of the patients received surgery or neoadjuvant therapy and had not started further treatment when recruited into this study.

### Statistical Analysis

Data entry, process, and analysis methods are the same as described previously (20, 21). Pearson's chi-square tests were used to compare the frequency distribution differences between women aged <50 years and ≥50 years. Mean and SD were calculated for all HRQoL domains. One-way ANOVA was used to calculate the *P*-value. The multivariate generalized linear model (GLM) was used to estimate all the factors with statistical difference to determine the features most strongly associated with these HRQoL scores. A two-sided *P*-value <0.05 was considered to be statistically significant.

## Results

**Figure 1:**
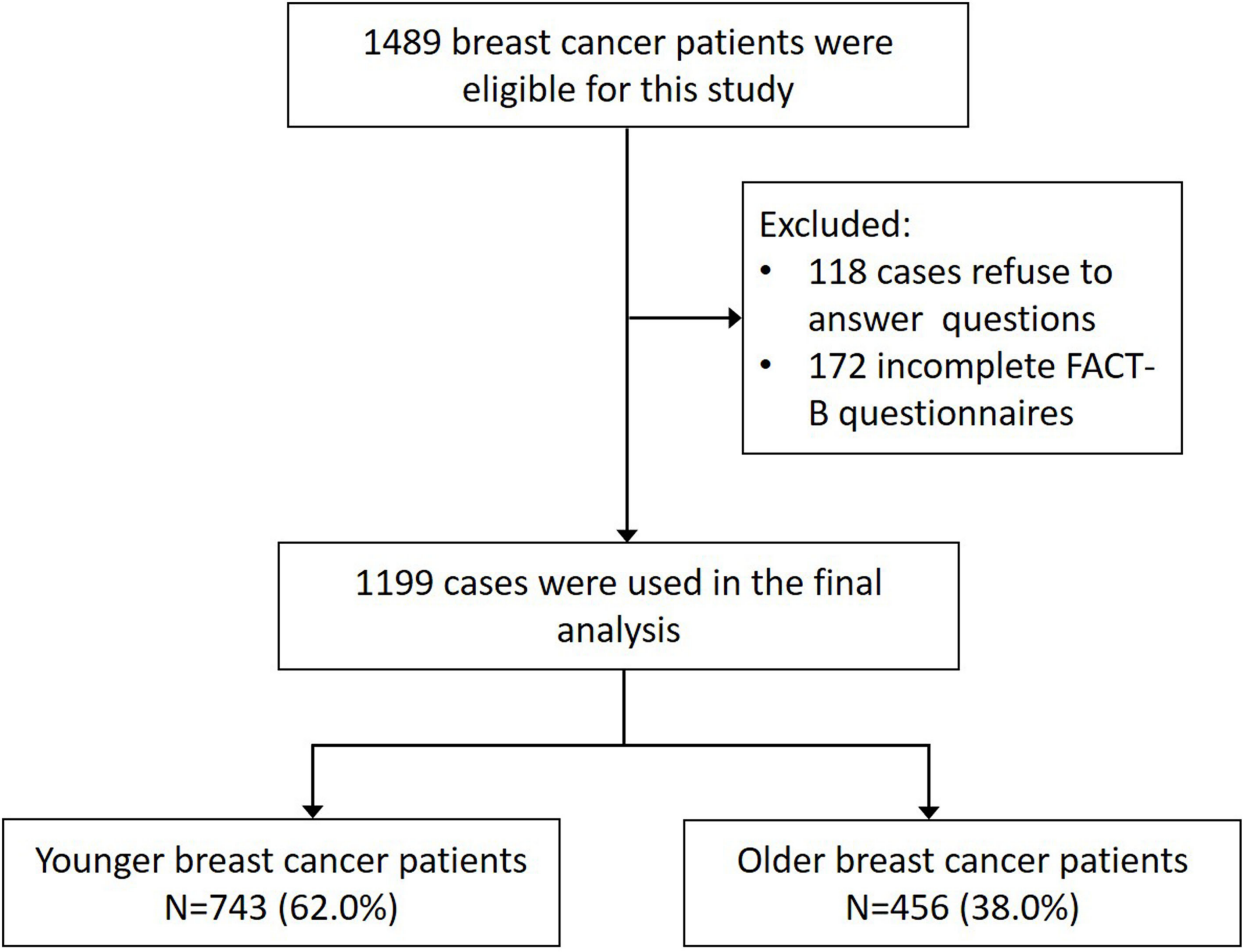
Flowchart of patients eligible for this study.

This study initially recruited 1,489 eligible patients with breast cancer as described previously, and 1,199 cases were used for analysis as appropriate. Of these women, the mean age was 47.66 years. In this study, the patients were divided into two groups: women aged <50 years and women aged ≥50 years. Women aged <50 years constituted 62.0% of the entire dataset. Figure 1 presents the flow of patients in the study. The basic characteristics of the two groups are shown in Table 1. There were statistically significant differences between the two groups in education levels (χ^2^ = 43.845, *P* < 0.001), family average revenue (χ^2^ = 11.962, *P* = 0.018), and post-menopausal status (χ^2^ = 585.054, *P* < 0.001). No significant differences were found for location, economic status, social status, marriage, and family history of breast cancer between different age groups. In this study, no significant lifestyle habits differences existed based on age categories, except for BMI distribution. Women aged <50 years reported significantly lower BMI (χ^2^ = 19.080, *P* < 0.001).

**Table 1:** Differences in the basic characteristics between younger and older breast cancer women.

**Variable**	**Age**		**χ^2^**	***P***
	**Younger (<50) (*n* = 743)**	**Older (≥50) (*n* = 456)**		
Basic demographic information				
Location			0.893	0.345
Urban	336 (47.6%)	225 (50.4%)		
Rural	370 (52.4%)	221 (49.6%)		
Education			43.845	<0.001
Elementary or low	102 (14.1%)	122 (27.9%)		
Middle	274 (38.0%)	126 (28.8%)		
High	220 (30.5%)	145 (33.1%)		
College	120 (16.6%)	44 (10.0%)		
Postgraduate	6 (0.8%)	1 (0.2%)		
Family average revenue (RMB)			11.962	0.018
<1,000	48 (6.6%)	47 (10.5%)		
1,000–1,999	119 (16.4%)	92 (20.6%)		
2,000–2,999	208 (28.7%)	117 (26.2%)		
3,000–4,999	175 (24.1%)	105 (23.5%)		
≥5,000	176 (24.2%)	85 (19.1%)		
Economic status			4.939	0.176
High	19 (2.6%)	13 (2.7%)		
Good	159 (21.8%)	97 (21.7%)		
Average	461 (63.2%)	262 (58.6%)		
Poor	90 (12.3%)	75 (16.8%)		
Social status			1.999	0.573
High	23 (3.2%)	13 (3.0%)		
Good	163 (22.5%)	103 (23.4%)		
Average	499 (68.9%)	292 (66.4%)		
Poor	39 (5.4%)	32 (7.3%)		
Menopause			585.054	<0.001
Yes	56 (7.7%)	340 (76.9%)		
No	667 (92.3%)	102 (23.1%)		
Marriage			1.376	0.241
Ever	722 (97.2%)	448 (98.2%)		
Never	21 (2.8%)	8 (1.8%)		
Family history of breast cancer			0.444	0.505
Yes	45 (6.3%)	23 (5.3%)		
No	668 (93.7%)	407 (94.7%)		
Lifestyle habits characteristics				
Cigarette smoking			1.465	0.226
Yes	20 (2.7%)	18 (4.0%)		
No	721 (97.3%)	436 (96.0%)		
Second-hand smoking			0.880	0.348
Yes	277 (60.5%)	174 (64.0%)		
No	181 (39.5%)	98 (36.0%)		
Alcohol drinking			0.109	0.742
Yes	96 (13.0%)	56 (12.3%)		
No	643 (87.0%)	398 (87.7%)		
Tea			1.640	0.200
Yes	141 (19.3%)	100 (22.4%)		
No	589 (80.7%)	346 (77.6%)		
Coffee			0.035	0.853
Yes	31 (4.3%)	20 (4.5%)		
No	694 (95.7%)	424 (95.5%)		
Sleep satisfaction			2.436	0.296
Very satisfied	90 (12.3%)	48 (10.8%)		
Satisfied	514 (70.4%)	305 (68.2%)		
Dissatisfied	126 (17.3%)	92 (20.7%)		
Current life satisfaction			4.565	0.102
Very satisfied	82 (11.0%)	39 (8.6%)		
Satisfied	493 (66.4%)	329 (72.1%)		
Dissatisfied	168 (22.6%)	88 (19.3%)		
Physical activity			2.142	0.343
Often	210 (28.5%)	129 (28.5%)		
Occasionally	311 (42.3%)	175 (38.6%)		
Never	215 (29.2%)	149 (32.9%)		
BMI (kg/m^2^)			19.080	<0.001
<24.0	373 (51.9%)	183 (42.6%)		
24.0–28.0	275 (38.2%)	169 (39.3%)		
>28.0	71 (9.9%)	78 (18.1%)		

The mean FACT-B scores for each individual HRQoL domain and overall scores are shown in Table 2. The mean FACT-B overall score of younger women was 83.63, while in the older women group, it was 85.88. Younger women (aged <50 years) at the diagnosis of breast cancer appeared to be related to lower scores than older women (aged ≥50 years) in all of the HRQoL subscales except PWB and SWB. EWB (*p* = 0.003), FWB (*p* = 0.006), BCS (*p* = 0.038), and FACT-B Total (*p* = 0.028) were significantly related to age.

**Table 2:** Health-related quality of life differences by age group.

	**Age**		***t***	***P***
	**Younger (<50) (*n* = 734)**	**Older (≥50) (*n* = 456)**		
PWB	19.45 ± 4.385	19.32 ± 4.798	0.217	0.614
SWB	16.14 ± 5.519	16.28 ± 5.214	0.182	0.670
EWB	13.92 ± 4.416	14.67 ± 4.475	8.623	0.003
FWB	12.80 ± 5.515	13.72 ± 5.746	7.656	0.006
BCS	21.32 ± 4.622	21.90 ± 4.849	4.337	0.038
Total	83.63 ± 16.211	85.88 ± 17.244	5.215	0.023

*PWB, physical well-being; SWB, social well-being; EWB, emotional well-being; FWB, functional well-being; BCS, breast cancer subscale (Higher scores represent better health-related quality of life)*.

As shown in Table 3, in order to more specifically examine which items in lifestyle habits were correlated with HRQoL among younger and older women diagnosed with breast cancer within 2 weeks, we compared the mean scores of the FACT-B Total between two age groups. The relationships of the nine lifestyle habit items with HRQoL were different between younger and older women. First, very satisfied with sleep and current life at diagnosis were criteria associated with higher scores in all the women with breast cancer. Second, tea (*p* = 0.009) and alcohol-drinking (*p* = 0.001) women showed a significantly higher score in the younger age group, while drinking coffee (*p* = 0.009) showed a significantly lower score in the older age group. Third compared to the younger age group, frequent participation in physical activities in the older age group was associated with higher HRQoL overall (*p* = 0.002). And smoking showed worse HRQoL in the older age group (*p* = 0.045). No other significant items of lifestyle habits were observed in association with FACT-B Total scores.

**Table 3:** Comparison of mean FACT-B Total scores of lifestyle habit items between the two age groups.

**Variable**	**Younger (<50)**			**Older (≥50)**		
	**Scores**	***t***	***P***	**Scores**	***t***	***P***
Cigarette smoking		3.838	0.050		4.026	0.045
Yes	91.10 ± 17.029			77.67 ± 15.080		
No	83.80 ± 16.418			85.79 ± 16.894		
Second-hand smoking		3.614	0.058		0.127	0.722
Yes	82.59 ± 16.531			85.61 ± 16.851		
No	85.67 ± 17.577			86.43 ± 18.928		
Alcohol drinking		10.498	0.001		0.191	0.662
Yes	89.06 ± 19.729			86.23 ± 17.958		
No	83.26 ± 15.820			85.17 ± 16.834		
Tea		6.788	0.009		3.775	0.053
Yes	87.22 ± 16.178			88.31 ± 18.895		
No	83.21 ± 16.510			84.55 ± 16.447		
Coffee		2.758	0.097		6.955	0.009
Yes	88.81 ± 18.541			75.50 ± 10.081		
No	83.77 ± 16.444			85.70 ± 17.150		
Sleep satisfaction		7.275	0.001		4.438	0.012
Very satisfied	89.63 ± 17.633			89.69 ± 18.659		
Satisfied	82.67 ± 15.894			83.68 ± 15.668		
Dissatisfied	84.89 ± 16.878			88.15 ± 18.804		
Current life satisfaction		11.823	<0.001		15.430	<0.001
Very satisfied	89.10 ± 19.505			93.05 ± 20.578		
Satisfied	84.75 ± 15.735			86.57 ± 16.390		
Dissatisfied	79.24 ± 15.840			77.41 ± 14.562		
Physical activity		2.915	0.055		6.345	0.002
Often	83.93 ± 17.145			89.51 ± 19.928		
Occasionally	85.53 ± 17.023			84.97 ± 16.064		
Never	82.01 ± 14.887			82.36 ± 14.589		
BMI (kg/m^2^)		1.991	0.137		1.435	0.239
<24.0	84.73 ± 16.417			85.97 ± 17.516		
24.0-28.0	82.25 ± 15.859			84.52 ± 15.862		
>28.0	84.82 ± 17.045			88.41 ± 17.180		

Meanwhile, we also analyzed the mean FACT-B scores for each individual HRQoL domain of the nine items in lifestyle habits in Supplementary Tables 1–5. Each individual domain with statistical differences is summarized in Table 4.

**Table 4:** The summary of each individual HRQoL domain with statistical differences in nine lifestyle habit items.

**Variable**	**Younger (<50)**	**Older (≥50)**
Cigarette smoking	SWB(+)	EWB(-)
Alcohol drinking	SWB(+), EWB(+), BCS(+)	
Tea	PWB(+), SWB(+), BCS(+)	BCS(+)
Coffee	PWB(+)	EWB(–), BCS(–)
Sleep satisfaction	PWB(+), SWB(+), EWB(+), FWB(+), BCS(+)	PWB(+), EWB(+), BCS(+)
Current life satisfaction	PWB(+), SWB(+), EWB(+), BCS(+)	PWB(+), SWB(+), EWB(+), FWB(+), BCS(+)
Physical activity	FWB(+)	PWB(+), EWB(+), BCS(+)
BMI (>28)		BCS(+)

*+: Higher score*.

*–: Lower score*.

*PWB, physical well-being; SWB, social well-being; EWB, emotional well-being; FWB, functional well-being; BCS, breast cancer subscale (Higher scores represent better health-related quality of life)*.

The relationship of lifestyle habits with HRQoL was further analyzed using GLM, as shown in Tables 5, 6. Tea and alcohol drinking, being very satisfied with sleep and with current life, were again the strongest predictors of higher HRQoL in the younger age group. Meanwhile, no coffee consumption, frequent participation in physical activities, and being very satisfied with sleep and current life were the key predictors of higher HRQoL in the older age group.

**Table 5:** Multivariable generalized linear models examining the health-related quality of life scores in relation to lifestyle habits in the younger group (aged <50 years).

	**B**	**S.E**.	**95% C.I**.		**Wald**	**df**	***P***
			**Lower**	**Upper**			
Alcohol drinking (yes)	6.030	1.7843	2.533	9.527	11.423	1	0.001
Tea (yes)	3.524	1.5218	0.541	6.507	5.363	1	0.021
Sleep satisfaction (very satisfied)	4.604	2.2634	0.168	9.040	4.137	1	0.042
Current life satisfaction (very satisfied)	9.376	2.2459	4.974	13.778	17.429	1	<0.001
Intercept	84.119	2.2748	79.661	88.578	1367.426	1	<0.001

**Table 6:** Multivariable generalized linear models examining the health-related quality of life scores in relation to lifestyle habits in the older group (aged ≥50 years).

	**B**	**S.E**.	**95% C.I**.		**Wald**	**df**	***P***
			**Lower**	**Upper**			
Coffee (no)	9.462	3.6877	16.609	2.234	6.583	1	0.010
Physical activity (often)	7.116	1.9780	3.239	10.993	12.943	1	<0.001
Sleep satisfaction (very satisfied)	4.095	1.9618	0.250	7.940	4.358	1	0.037
Current life satisfaction (very satisfied)	15.438	3.2623	8.954	21.742	22.134	1	<0.001
Intercept	78.109	4.2783	69.724	86.494	333.311	1	<0.001

## Discussion

In this study, we examined the HRQoL among patients with breast cancer in Chinese populations and investigated their relationship with specific lifestyle habits factors. To the best of our knowledge, this is the first report on the relationship between ages and the specific lifestyle habits factors and HRQoL in Chinese patients with breast cancer using the FACT-B questionnaires. The FACT-B is an international scale developed by Rush-Presbyterian-St. Luke's Medical Center, Chicago, United States, which is widely used to assess the HRQoL of patients with breast cancer (22). It has been translated into many languages, such as (simplified) Chinese, Malayalam, and Korean (23). Previous studies have demonstrated that Chinese versions of the FACT-B (version 4) are effective, sensitive, and reliable in evaluating the HRQoL of patients with breast cancer in China (23–25), which was also confirmed in this study. Using this internationally consistent and effective scale to assess the QoL of Chinese patients is important to improve our understanding of the prognosis of breast cancer.

Previous studies have mostly performed HRQoL tests on female patients for at least 4 months after a breast cancer diagnosis (12, 13, 15). By this time, these patients with breast cancer have begun their initial treatment process and have had some time to adapt to their condition. There are few studies that conducted HRQoL tests within a few weeks of a woman being newly diagnosed with breast cancer (19). In this study, all the newly diagnosed patients have not received surgery or neoadjuvant therapy and have not started further treatment when recruited into this study. As previous studies have demonstrated, the diagnosis of cancer will directly impact a person's mental health immediately, and the ability to cope with the diagnosis will subsequently change greatly (13, 26). The early psychosocial adaptation to a diagnosis of breast cancer may have important effects on some survivorship issues, such as receiving and adhering to treatment (27, 28), coping mechanisms (28), and long-term prognosis (29, 30). In this study, 1,199 patients came from 22 different hospitals, and the formulation and decision-making process of each patient's treatment plan was different. Therefore, the completion time of FACT-B questionnaire ranged from 1 day to 14 days after diagnosis. Hence, we set the time point as within 2 weeks of breast cancer diagnosis to analyze the early factors affecting HRQoL of patients with breast cancer in China.

Moreover, the age group with the highest incidence of breast cancer in women is 45–59 years old, and some independent studies have reported that the peak age of breast cancer was between 45 and 55 years in China (3, 20). In this study, as an approximate indicator of menopausal status, we divided the age into two groups of <50 years old and ≥50 years old; this cutoff point was used for epidemiological studies and HRQoL studies of large-scale breast cancer as well as clinical practice. The results of this study suggested that older patients with breast cancer showed better HRQoL than younger women in most of the HRQoL domains except SWB and PWB, which was supported in previous studies (31, 32). Compared to older women, the younger women are more susceptible to suffering from psychosocial influences (13, 31), may receive more aggressive treatment than older groups, and are more likely to receive chemotherapy, while older patients have more resources or skills to deal with breast cancer and maintain economic stability.

This study complements the very limited number of research studies that access the impact of lifestyle habits [i.e., smoking (including passive smoking), alcohol intake, dietary habits (tea and coffee), sleep satisfaction, current life satisfaction, physical activity, and BMI] on HRQoL in Chinese women who were diagnosed with breast cancer within 2 weeks. The results suggest that patients with breast cancer who adopted different lifestyle behaviors had different HRQoL between ages, as shown in Table 3 and Supplementary Tables 1–5. In this study, cigarette smoking in younger women showed a better SWB while being associated with worse EWB in the older age group, and alcohol drinking was significantly related to better SWB, EWB, and BCS in the younger age group. Previous studies have demonstrated that women who drunk more alcohol daily reported fewer disturbing vasomotor symptoms (33), which are considered to be the most specific symptom of menopause (34), while women who smoked cigarettes daily had more symptoms of depression than non-smokers, except for the menstrual symptoms domain (33, 35). These observations are in agreement with the results of this study, which show worse EWB in older smokers and high HRQoL (including SWB, EWB, and BCS) in younger alcohol consumers.

Clinically significant premenstrual syndrome (PMS) affects 15–20% of premenopausal women and significantly reduces QoL (36). Rossignol et al. (37, 38), along with three other similar studies (39, 40), found a strong positive correlation between caffeine and coffee intake and premenstrual syndrome. Women with severe premenstrual symptoms appear to be able to alter caffeine intake, increasing caffeine intake to treat symptoms such as fatigue. These observations support our result that younger women with tea and coffee intake have higher HRQoL (87.22 and 88.81, respectively). In contrast, tea in China has thousands of years of cultural heritage, and older Chinese women prefer to drink traditional tea but refuse coffee, which causes no coffee to have a higher HRQoL. However, this explanation should be provided with discretion and needs more study. But it is worth noting that tea intake was associated with higher HRQoL in both younger and older women. Some studies have shown that tea or its constituents (41), particularly, epigallocatechin-3-gallate as the most abundant and biologically active tea catechins (42), suppress mammary tumorigenesis *via* effects on antioxidant activity (43), sex hormones (44), or different molecular pathways (45), which may also have a potential impact on QoL.

It is important to note that the frequency of physical activity was positively associated with higher HRQoL of patients with breast cancer in older group, while there was no statistical difference in the younger group. Angenete et al. found that the preoperative physical activity is positively associated with an enhanced physical recovery after breast cancer surgery (46). More importantly, evidence from observational studies shows a statistically significant positive correlation between inactivity and sedentary behavior and breast cancer risk and poorer health outcomes (47, 48). In patients with breast cancer, higher levels of physical activity have been shown to be associated with fewer adverse treatment-related side effects, higher HRQoL, and improved disease-specific prognoses, including longer survival and reduced risk of recurrence and mortality (16, 49). With this evidence, guidelines for physical activity for breast cancer survivors recommend that physical activity should be an integral and ongoing part of the care of all patients with breast cancer.

A population-based survey of HRQoL conducted by Katainen et al. suggested that BMI was a risk factor for lower HRQoL (33). Women with normal BMI had more physical and vasomotor symptoms than women with BMI lower than 25 kg/m^2^. Women with BMI higher than 30 kg/m^2^ had more physical and depressive symptoms than women with a BMI lower than 25 kg/m^2^ and have more cognitive impairments than women with lower BMI. In our study, though the distribution of BMI between the younger and older women in China with breast cancer showed significant differences, the BMI showed no influence on HRQoL in all the women. The remaining factors, very satisfied with sleep and current life at diagnosis, were associated with higher HRQoL scores in all the women with breast cancer, as expected.

Finally, the inherent limitations of this study should not be neglected. The most important limitations of this study include that the study was descriptive and cross-sectional, and some factors were collected retrospectively, which may have influenced our results; in our study, all the newly diagnosed patients were enrolled to complete the FACT-B questionnaire before receiving surgery or neoadjuvant therapy and further treatment, so the exact stage of the disease has not been determined, which may be an important factor affecting the HRQoL of patients. Notwithstanding its limitations, the results of this large population-based study may help guide interventions to improve QoL. It is believed that this study can provide reference and a basis for future research and also pave the road for finding patient-centered solutions for evidence-based selection of optimal treatments, decision-making process, psychosocial interventions, patient–physician communications, etc.

## Conclusion

In summary, we found that younger patients <50 years showed significantly lower HRQoL than older patients ≥50 years. The relationship of the nine lifestyle habit items with HRQoL was different between younger and older women. The associated variable of QoL can help clinicians identify patients at risk for low QoL. When these characteristics or situations can be balanced, changing them through intervention can improve a patient's QoL, and as women gradually receive treatment and then enter into their long-term survivorship period, their effects may change subsequent adjustments and functions regarding breast cancer, consequently improving the prognosis of patients with breast cancer.

## Data Availability Statement

The original contributions presented in the study are included in the article/Supplementary Material, further inquiries can be directed to the corresponding author/s.

## Ethics Statement

All procedures performed involving human participants were in accordance with the Ethical Standards of the Second Hospital of Shandong University Research Committee with the approval number 2010004. The patients/participants provided their written informed consent to participate in this study.

## Author Contributions

Z-GY conceived the study. CZ, H-YJ, and LL contributed to the study design and performed statistical analyses. CZ and LL wrote the manuscript. L-YL, FW, FZ, Y-JX, M-MG, Y-JW, and S-YH contributed to manuscript revision and statistical analyses. S-DC, F-GT, Z-MF, C-ZG, X-CC, Z-LY, XW, HL, SW, H-CJ, X-ND, H-BW, G-LL, Q-TW, J-GZ, FJ, J-HT, LL, S-GZ, and W-SZ contributed to the collection of the data and biological samples. All authors contributed to the article and approved the submitted version.

## Funding

This research was primarily granted funding from the Minister-affiliated hospital key project of the Ministry of Health of the People's Republic of China (establishment and improvement of high-risk populations screening and evaluation system for breast cancer), the Major Scientific and Technological Innovation Project of Shandong Province (2017CXGC1212), the National Key Research and Development Program of China (2016YFC0901304), the National Natural Science Foundation of China (81702603), the Taishan Scholars Program of Shandong Province (tsqn201812135), and the China Postdoctoral Science Foundation (2021T140408 and 2021M691934).

## Conflict of Interest

The authors declare that the research was conducted in the absence of any commercial or financial relationships that could be construed as a potential conflict of interest.

## Publisher's Note

All claims expressed in this article are solely those of the authors and do not necessarily represent those of their affiliated organizations, or those of the publisher, the editors and the reviewers. Any product that may be evaluated in this article, or claim that may be made by its manufacturer, is not guaranteed or endorsed by the publisher.

## Abbreviations

HRQoL: Health-related quality of life
WHO: World Health Organization
QoL: Quality of life
FACT-B: Functional Assessment of Cancer Therapy-Breast Cancer
BCS: Breast cancer subscale
PWB: Physical well-being
SWB: Social/family well-being
EWB: Emotional well-being
FWB: Functional well-being
PMS: Premenstrual syndrome
ANOVA: Analysis of variance.
