# Supplementary material for: Relationship Between Lifestyle Habits and Health-Related Quality of Life of Recently Diagnosed Breast Cancer Patients: A Comparison Between Younger and Older Women in China
Source: Front Public Health. 2021 Dec 14;9:767151. doi: 10.3389/fpubh.2021.767151 (PMC8714764; doi:10.3389/fpubh.2021.767151)
Supplement: Supplementary file 1 [file Table_1.docx]

**Relationship Between Lifestyle Habits and Health-Related Quality of life of Recently Diagnosed Breast Cancer Patients: A Comparison Between Younger and Older Women in China.**

We analyzed the mean FACT-B scores for each individual HRQoL domain of the 9 items in lifestyle habits in ESM_1-5.

Cigarette smoking in younger women showed a better SWB (p=0.009), while associated with worse EWB in older age group (p=0.040).

Alcohol drinking was significantly related to better SWB, EWB and BCS in younger age group (p=0.001, 0.001, 0.042, respectively), but the older age group did not show any correlation.

Tea consumption in younger women showed higher PWB, SWB and BCS scores (p=0.001, 0.012, 0.049, respectively), while older women showed only higher BCS scores (p=0.001). Opposite to the better PWB of coffee consumption in younger age group (p=0.019), the older group showed worse EWB and BCS (p= 0.008 and 0.011).

Sleep satisfaction in younger women was associated with higher scores in all individual HRQoL domain, in contrast, current life satisfaction in older women showed the similar results.

In addition, frequent participation in physical activities in older age group is associated with better PWB, EWB and BCS (p=0.021, 0.001, 0.002, respectively).

Second-hand smoking and BMI distribution group did not show any correlation to each individual HRQoL domain, except for BMI ＞28.0 in older age group that showed better BCS (p=0.019).

**Electronic Supplementary Material**

Supplementary Table 1 Comparison of mean physical well being (PWB) scores of lifestyle habit items between the two age groups.

| Variable | Younger(＜50) | | | Older(≥50) | | |
| --- | --- | --- | --- | --- | --- | --- |
|  | Scores | F | P | Scores | F | P |
| **Cigarette smoking** |  | 2.380 | 0.123 |  | 2.982 | 0.085 |
| Yes | 21.00±5.109 |  |  | 17.39±5.158 |  |  |
| No | 19.47±4.366 |  |  | 19.36±4.717 |  |  |
| **Second-hand smoking** |  | 0.213 | 0.644 |  | 0.032 | 0.859 |
| Yes | 19.30±4.498 |  |  | 19.41±5.201 |  |  |
| No | 19.50±4.554 |  |  | 19.30±4.600 |  |  |
| **Alcohol drinking** |  | 1.845 | 0.175 |  | 1.176 | 0.279 |
| Yes | 20.07±4.861 |  |  | 19.88±5.138 |  |  |
| No | 19.42±4.320 |  |  | 19.13±4.743 |  |  |
| **Tea** |  | 10.947 | 0.001 |  | 1.361 | 0.244 |
| Yes | 20.60±4.163 |  |  | 19.72±5.027 |  |  |
| No | 19.24±4.442 |  |  | 19.08±4.766 |  |  |
| **Coffee** |  | 5.552 | 0.019 |  | 3.168 | 0.076 |
| Yes | 21.32±4.693 |  |  | 17.35±4.221 |  |  |
| No | 19.42±4.394 |  |  | 19.31±4.835 |  |  |
| **Sleep satisfaction** |  | 9.956 | ＜0.001 |  | 4.298 | 0.014 |
| Very satisfied | 21.37±3.993 |  |  | 20.58±4.114 |  |  |
| Satisfied | 19.16±4.372 |  |  | 18.82±4.836 |  |  |
| Dissatisfied | 19.54±4.436 |  |  | 19.98±4.565 |  |  |
| **Current life satisfaction** |  | 18.917 | ＜0.001 |  | 14.047 | ＜0.001 |
| Very satisfied | 20.94±4.071 |  |  | 19.85±4.804 |  |  |
| Satisfied | 19.83±4.124 |  |  | 19.80±4.557 |  |  |
| Dissatisfied | 17.82±4.844 |  |  | 16.88±4.957 |  |  |
| **Physical activity** |  | 1.591 | 0.205 |  | 3.899 | 0.021 |
| Often | 19.42±4.485 |  |  | 19.68±5.059 |  |  |
| Occasionally | 19.85±4.205 |  |  | 19.67±4.476 |  |  |
| Never | 19.17±4.540 |  |  | 18.34±4.846 |  |  |
| **BMI (kg/m**2**)** |  | 1.942 | 0.144 |  | 2.291 | 0.102 |
| ＜24.0 | 19.65±4.376 |  |  | 19.25±4.993 |  |  |
| 24.0-28.0 | 19.06±4.305 |  |  | 18.92±4.460 |  |  |
| ＞28.0 | 19.94±4.834 |  |  | 20.29±4.542 |  |  |

Supplementary Table 2 Comparison of mean social well being (SWB) scores of lifestyle habit items between the two age groups.

| Variable | Younger(＜50) | | | Older(≥50) | | |
| --- | --- | --- | --- | --- | --- | --- |
|  | Scores | F | P | Scores | F | P |
| **Cigarette smoking** |  | 6.824 | 0.009 |  | 0.373 | 0.542 |
| Yes | 19.20±5.156 |  |  | 15.44±4.204 |  |  |
| No | 16.14±5.170 |  |  | 16.20±5.190 |  |  |
| **Second-hand smoking** |  | 0.826 | 0.364 |  | 0.327 | 0.568 |
| Yes | 16.48±4.829 |  |  | 16.43±5.287 |  |  |
| No | 16.91±5.175 |  |  | 16.82±5.423 |  |  |
| **Alcohol drinking** |  | 12.092 | 0.001 |  | 0.729 | 0.394 |
| Yes | 17.94±5.494 |  |  | 16.70±5.985 |  |  |
| No | 15.98±5.097 |  |  | 16.07±5.059 |  |  |
| **Tea** |  | 6.396 | 0.012 |  | 1.555 | 0.213 |
| Yes | 17.20±5.091 |  |  | 16.73±5.610 |  |  |
| No | 15.97±5.206 |  |  | 16.01±4.964 |  |  |
| **Coffee** |  | 0.809 | 0.369 |  | 1.306 | 0.254 |
| Yes | 17.03±4.841 |  |  | 14.75±5.637 |  |  |
| No | 16.17±5.228 |  |  | 16.11±5.164 |  |  |
| **Sleep satisfaction** |  | 7.236 | 0.001 |  | 1.595 | 0.204 |
| Very satisfied | 16.83±6.277 |  |  | 16.44±5.849 |  |  |
| Satisfied | 15.77±4.997 |  |  | 15.86±4.813 |  |  |
| Dissatisfied | 17.61±4.777 |  |  | 16.91±5.712 |  |  |
| **Current life satisfaction** |  | 3.983 | 0.019 |  | 5.243 | 0.006 |
| Very satisfied | 17.66±6.084 |  |  | 18.62±5.245 |  |  |
| Satisfied | 16.16±5.073 |  |  | 16.01±5.177 |  |  |
| Dissatisfied | 15.73±4.946 |  |  | 15.55±4.868 |  |  |
| **Physical activity** |  | 0.021 | 0.980 |  | 1.596 | 0.204 |
| Often | 16.25±4.879 |  |  | 16.81±5.514 |  |  |
| Occasionally | 16.21±5.632 |  |  | 16.06±5.160 |  |  |
| Never | 16.30±4.823 |  |  | 15.71±4.881 |  |  |
| **BMI (kg/m**2**)** |  | 1.871 | 0.155 |  | 1.165 | 0.313 |
| ＜24.0 | 16.46±5.313 |  |  | 16.63±5.239 |  |  |
| 24.0-28.0 | 15.74±4.862 |  |  | 15.90±4.872 |  |  |
| ＞28.0 | 16.70±5.829 |  |  | 15.78±5.830 |  |  |

Supplementary Table 3 Comparison of mean emotional well being (EWB) scores of lifestyle habit items between the two age groups.

| Variable | Younger(＜50) | | | Older(≥50) | | |
| --- | --- | --- | --- | --- | --- | --- |
|  | Scores | F | P | Scores | F | P |
| **Cigarette smoking** |  | 2.182 | 0.140 |  | 4.263 | 0.040 |
| Yes | 15.35±3.360 |  |  | 12.44±4.382 |  |  |
| No | 13.96±4.179 |  |  | 14.66±4.641 |  |  |
| **Second-hand smoking** |  | 3.276 | 0.071 |  | 0.417 | 0.519 |
| Yes | 13.54±4.377 |  |  | 15.05±4.978 |  |  |
| No | 14.29±4.262 |  |  | 14.65±4.717 |  |  |
| **Alcohol drinking** |  | 11.919 | 0.001 |  | 0.191 | 0.662 |
| Yes | 15.34±5.309 |  |  | 14.93±5.109 |  |  |
| No | 13.78±3.930 |  |  | 14.51±4.379 |  |  |
| **Tea** |  | 3.703 | 0.055 |  | 2.816 | 0.094 |
| Yes | 14.60±4.357 |  |  | 15.22±5.090 |  |  |
| No | 13.85±4.100 |  |  | 14.37±4.285 |  |  |
| **Coffee** |  | 1.493 | 0.222 |  | 7.043 | 0.008 |
| Yes | 14.90±4.700 |  |  | 12.00±4.460 |  |  |
| No | 13.97±4.137 |  |  | 14.70±4.442 |  |  |
| **Sleep satisfaction** |  | 7.025 | 0.001 |  | 5.416 | 0.005 |
| Very satisfied | 15.30±4.172 |  |  | 15.85±4.654 |  |  |
| Satisfied | 13.62±4.035 |  |  | 14.12±4.336 |  |  |
| Dissatisfied | 14.35±4.482 |  |  | 15.47±4.713 |  |  |
| **Current life satisfaction** |  | 10.582 | ＜0.001 |  | 12.794 | ＜0.001 |
| Very satisfied | 14.95±4.619 |  |  | 15.67±5.303 |  |  |
| Satisfied | 14.24±4.099 |  |  | 15.01±4.359 |  |  |
| Dissatisfied | 12.77±3.869 |  |  | 12.50±3.883 |  |  |
| **Physical activity** |  | 1.210 | 0.299 |  | 7.607 | 0.001 |
| Often | 13.67±4.283 |  |  | 15.84±4.911 |  |  |
| Occasionally | 14.24±3.898 |  |  | 14.25±3.995 |  |  |
| Never | 13.94±4.440 |  |  | 13.88±4.419 |  |  |
| **BMI (kg/m**2**)** |  | 1.124 | 0.325 |  | 2.049 | 0.130 |
| ＜24.0 | 14.04±4.196 |  |  | 14.39±4.469 |  |  |
| 24.0-28.0 | 13.62±4.021 |  |  | 14.57±4.417 |  |  |
| ＞28.0 | 14.27±4.263 |  |  | 15.58±4.281 |  |  |

Supplementary Table 4 Comparison of mean functional well being (FWB) scores of lifestyle habit items between the two age groups.

| Variable | Younger(＜50) | | | Older(≥50) | | |
| --- | --- | --- | --- | --- | --- | --- |
|  | Scores | F | P | Scores | F | P |
| **Cigarette smoking** |  | 0.345 | 0.557 |  | 2.385 | 0.123 |
| Yes | 13.60±5.175 |  |  | 11.61±4.840 |  |  |
| No | 12.86±5.588 |  |  | 13.71±5.677 |  |  |
| **Second-hand smoking** |  | 2.436 | 0.119 |  | 0.346 | 0.557 |
| Yes | 12.34±5.194 |  |  | 12.87±5.212 |  |  |
| No | 13.17±6.063 |  |  | 13.30±6.226 |  |  |
| **Alcohol drinking** |  | 0.970 | 0.325 |  | 1.687 | 0.195 |
| Yes | 13.42±6.574 |  |  | 12.66±6.573 |  |  |
| No | 12.82±5.406 |  |  | 13.71±5.528 |  |  |
| **Tea** |  | 0.121 | 0.729 |  | 0.194 | 0.660 |
| Yes | 12.72±5.766 |  |  | 13.44±5.962 |  |  |
| No | 12.90±5.543 |  |  | 13.72±5.566 |  |  |
| **Coffee** |  | 1.029 | 0.311 |  | 1.131 | 0.288 |
| Yes | 13.84±7.244 |  |  | 12.25±6.640 |  |  |
| No | 12.80±5.512 |  |  | 13.64±5.663 |  |  |
| **Sleep satisfaction** |  | 3.750 | 0.024 |  | 0.547 | 0.579 |
| Very satisfied | 13.57±6.084 |  |  | 13.77±6.501 |  |  |
| Satisfied | 13.04±5.453 |  |  | 13.59±5.155 |  |  |
| Dissatisfied | 11.71±5.469 |  |  | 12.95±6.444 |  |  |
| **Current life satisfaction** |  | 0.260 | 0.771 |  | 5.063 | 0.007 |
| Very satisfied | 13.15±5.934 |  |  | 16.33±5.993 |  |  |
| Satisfied | 12.78±5.688 |  |  | 13.31±5.748 |  |  |
| Dissatisfied | 13.07±5.041 |  |  | 13.48±4.980 |  |  |
| **Physical activity** |  | 6.360 | 0.002 |  | 1.256 | 0.286 |
| Often | 11.77±4.918 |  |  | 14.12±6.609 |  |  |
| Occasionally | 13.49±6.069 |  |  | 13.72±5.669 |  |  |
| Never | 13.14±5.365 |  |  | 13.05±5.360 |  |  |
| **BMI (kg/m**2**)** |  | 0.397 | 0.672 |  | 0.610 | 0.544 |
| ＜24.0 | 13.06±5.684 |  |  | 14.19±5.929 |  |  |
| 24.0-28.0 | 12.77±5.086 |  |  | 13.61±4.977 |  |  |
| ＞28.0 | 12.54±6.134 |  |  | 13.55±5.978 |  |  |

Supplementary Table 5 Comparison of mean breast cancer subscale (BCS) scores of lifestyle habit items between the two age groups.

| Variable | Younger(＜50) | | | Older(≥50) | | |
| --- | --- | --- | --- | --- | --- | --- |
|  | Scores | F | P | Scores | F | P |
| **Cigarette smoking** |  | 0.290 | 0.591 |  | 0.877 | 0.349 |
| Yes | 21.95±5.871 |  |  | 20.78±4.821 |  |  |
| No | 21.38±4.612 |  |  | 21.86±4.821 |  |  |
| **Second-hand smoking** |  | 3.700 | 0.055 |  | 0.167 | 0.683 |
| Yes | 20.92±4.720 |  |  | 21.98±5.034 |  |  |
| No | 21.80±4.770 |  |  | 22.24±4.928 |  |  |
| **Alcohol drinking** |  | 4.137 | 0.042 |  | 0.211 | 0.646 |
| Yes | 22.29±5.500 |  |  | 22.07±4.935 |  |  |
| No | 21.26±4.495 |  |  | 21.75±4.828 |  |  |
| **Tea** |  | 3.882 | 0.049 |  | 11.457 | 0.001 |
| Yes | 22.10±4.158 |  |  | 23.20±5.041 |  |  |
| No | 21.24±4.744 |  |  | 21.38±4.649 |  |  |
| **Coffee** |  | 0.122 | 0.727 |  | 6.537 | 0.011 |
| Yes | 21.71±4.474 |  |  | 19.15±3.438 |  |  |
| No | 21.41±4.666 |  |  | 21.95±4.839 |  |  |
| **Sleep satisfaction** |  | 4.309 | 0.014 |  | 5.625 | 0.004 |
| Very satisfied | 22.57±4.738 |  |  | 23.04±4.877 |  |  |
| Satisfied | 21.08±4.580 |  |  | 21.29±4.737 |  |  |
| Dissatisfied | 21.68±4.776 |  |  | 22.85±4.772 |  |  |
| **Current life satisfaction** |  | 12.877 | ＜0.001 |  | 19.682 | ＜0.001 |
| Very satisfied | 22.40±4.924 |  |  | 22.59±5.566 |  |  |
| Satisfied | 21.74±4.450 |  |  | 22.45±4.516 |  |  |
| Dissatisfied | 19.86±4.750 |  |  | 19.01±4.657 |  |  |
| **Physical activity** |  | 2.155 | 0.115 |  | 6.214 | 0.002 |
| Often | 20.89±4.464 |  |  | 23.07±5.118 |  |  |
| Occasionally | 21.75±4.625 |  |  | 21.27±4.884 |  |  |
| Never | 21.38±4.835 |  |  | 21.38±4.336 |  |  |
| **BMI (kg/m**2**)** |  | 0.745 | 0.475 |  | 3.992 | 0.019 |
| ＜24.0 | 21.51±4.702 |  |  | 21.50±4.470 |  |  |
| 24.0-28.0 | 21.06±4.429 |  |  | 21.52±4.940 |  |  |
| ＞28.0 | 21.37±4.923 |  |  | 23.21±4.610 |  |  |
